# iPSC-derived exosomes as amphotericin B carriers: a promising approach to combat cryptococcal meningitis

**DOI:** 10.3389/fmicb.2025.1531425

**Published:** 2025-02-10

**Authors:** Jingyu Zhao, Wei Fang, Yangjie Gao, Jiquan Chen, Guizhen Wang, Julin Gu

**Affiliations:** ^1^Department of Dermatology, Third Affiliated Hospital of Naval Medical University, Shanghai, China; ^2^Department of Dermatology, The Third Affiliated Hospital of Soochow University, Changzhou, China; ^3^Department of Laser and Aesthetic Medicine, Shanghai Ninth People's Hospital, Shanghai JiaoTong University School of Medicine, Shanghai, China; ^4^Department of Pulmonary and Critical Care Medicine, Third Affiliated Hospital of Naval Medical University, Shanghai, China; ^5^Department of Emergency, Shanghai Tenth People’s Hospital, School of Medicine Tongji University, Shanghai, China

**Keywords:** *Cryptococcus neoformans*, cryptococcal meningitis, amphotericin B, induced pluripotent stem cells, drug delivery systems

## Abstract

**Background:**

Cryptococcal meningitis (CM) is a significant global health issue, particularly affecting individuals with HIV. Amphotericin B (AmB) serves as the cornerstone treatment for CM; however, its clinical application is restricted due to limited penetration of the blood–brain barrier and associated nephrotoxicity.

**Objective:**

This study investigates the use of exosomes derived from induced pluripotent stem cells (iPSC-Exos) as carriers for AmB in treating CM, aiming to enhance therapeutic efficacy and safety and reduce AmB toxicity.

**Methods:**

Exosomes were extracted from iPSC culture supernatants using ultrafiltration and ultracentrifugation. Their morphology and size were analyzed using transmission electron microscopy (TEM) and nanoparticle flow cytometry (nFCM). Purity was confirmed by Western blotting for markers CD9, CD63, and TSG101. AmB was loaded into iPSC-Exos using a co-incubation method. The cytotoxicity of the iPSC-Exo/AmB complex was evaluated on HEK 293 T and RAW264.7 cells using the CCK-8 assay, while apoptosis was assessed using live/dead cell staining and flow cytometry. The hemolytic effects were tested using rabbit red blood cells. In a C57BL/6 J mouse model of cryptococcal infection, treatment groups (AmB, iPSC-Exo/AmB, and iPSC-Exo) were administered corresponding drugs, with blood and brain samples collected for analysis. The minimum inhibitory concentration (MIC) of iPSC-Exo/AmB and conventional AmB against *Cryptococcus* was determined.

**Results:**

The iPSC-Exo/AmB complex exhibited reduced cytotoxicity *in vitro* and decreased AmB-induced renal and hepatic toxicity *in vivo*. Its MIC against *Cryptococcus* was over eight times lower than conventional AmB, significantly reducing fungal burden in the mouse brain and lowering serum inflammatory factors.

**Conclusion:**

The iPSC-Exo/AmB complex is a promising therapeutic strategy that enhances AmB efficacy while reducing toxicity, offering new hope for treating CM and other refractory fungal infections of the central nervous system.

## 1 Introduction

*Cryptococcus* is a clinically significant pathogenic fungus that preferentially infects the central nervous system (CNS), leading to cryptococcal meningitis (CM), a disease characterized by a high mortality rate (May et al., 2016; Tugume et al., 2023). CM is the most prevalent form of fungal meningitis globally, particularly among individuals with HIV. The estimated annual incidence of CM is approximately 278,000 cases, resulting in up to 181,000 deaths. As a result, it has become the second leading cause of HIV-related deaths worldwide (Hamed et al., 2023).

Amphotericin B (AmB) is a cornerstone antifungal drug that exerts its potent antifungal effect by binding to ergosterol in the fungal cell membrane, forming pores that lead to the leakage of intracellular substances (Wang et al., 2021). Although AmB is highly selective for fungal cells, its interaction with cholesterol in the host cell membrane results in significant host toxicity, particularly nephrotoxicity, which limits its clinical application (McHale et al., 2023). In addition, AmB’s ability to treat brain fungal infections, such as CM, is compromised by its limited penetration of the blood–brain barrier (BBB), affecting its therapeutic efficacy (Hamill, 2013; Wang et al., 2021).

Exosomes are key mediators of intercellular communication and have emerged as promising candidates for treating CNS diseases due to their non-tumorigenic nature, low immunogenicity, and ability to cross the BBB (Pegtel and Gould, 2019; Liang et al., 2021; Wu et al., 2021). Exosomes derived from induced pluripotent stem cells (iPSC-Exos) are particularly promising in CNS therapy due to their unique biological characteristics (Kolagar et al., 2020; Pasteuning-Vuhman et al., 2021; Ma et al., 2024). This study explores the use of iPSC-Exos as carriers for AmB (iPSC-Exo/AmB) to reduce drug toxicity and enhance its penetration of the BBB, thereby improving its therapeutic efficacy against CM.

## 2 Methods

### 2.1 Reagents

AmB was purchased from MedChemExpress (New Jersey, United States). Rabbit monoclonal anti-CD9 and anti-CD63 antibodies were obtained from CST (MA, United States). Recombinant anti-TSG101 and anti-calnexin antibodies were sourced from Abcam (Cambridge, UK). Goat anti-rabbit IgG was obtained from Zhongshan Golden Bridge Biotechnology (Guangdong, China).

### 2.2 Strains and cells

The standard strains of *Candida parapsilosis* ATCC 22019, wild-type (WT) H99 strains of *Cryptococcus neoformans,* and clinical isolates of *Cryptococcus neoformans* were obtained from the Shanghai Key Laboratory of Molecular Medical Mycology. All strains were cultured in yeast peptone dextrose (YPD) liquid medium (1% yeast extract, 2% peptone, and 2% glucose) in a shaking incubator at 30°C with 200 rpm.

Induced pluripotent stem cells (iPSCs) were obtained from the Changzhou Xitaihu Institute for Frontier Technology of Cell Therapy and cultured in StemFlex^™^ Basal Medium supplemented with 10× StemFlex^™^ additives and 1% penicillin–streptomycin (Gibco, Thermo Fisher Scientific, USA). For exosome collection, DMEM/F12 supplemented with GDEV^™^ Medium Supplement 10× (Guodian Pharmaceutical, Beijing, China) was used. Human embryonic kidney cells (HEK 293 T) and RAW264.7 macrophages were obtained from the Cell Bank of the Chinese Academy of Sciences and cultured in Dulbecco’s Modified Eagle Medium (DMEM) with 10% (v/v) heat-inactivated fetal bovine serum (FBS) (Gibco, Thermo Fisher Scientific, USA). All cell types were maintained at 37°C in a 5% CO2 atmosphere.

### 2.3 Mice

C57BL/6 J mice were obtained from Shanghai Yishang Biotechnology. All animal experiments were approved by the Institutional Animal Care and Use Committee (IACUC) (IACUC-2023-Mi-211) and conducted in accordance with the “Regulations on the Administration of Laboratory Animals” approved by the State Council of the People’s Republic of China.

### 2.4 Extraction and identification of iPSC-Exos

Initially, the collected iPSC supernatant was centrifuged at 3000 *g* for 10 min to remove cell debris. Next, the supernatant was filtered using a 0.22-μm filter to sterilize and collect the filtrate. The filtrate was subsequently added to a 100 kDa ultrafiltration tube (Millipore, MA, United States) and centrifuged at 3000 *g* at 4°C for concentration and purification until all filtrate was processed. Following this, ultracentrifugation was conducted at 100,000 *g* at 4°C for 90 min, resulting in the precipitation of exosomes at the bottom of the centrifuge tube. Finally, the supernatant was carefully removed to avoid disturbing the pellet, and the pellet was gently resuspended in PBS to obtain the exosome suspension.

We used the Western blot method to identify exosomes by sequentially loading a pre-stained protein ladder (Thermo Fisher Scientific, Waltham, MA, United States), iPSC exosomal protein samples, and control iPSC protein samples (prepared via RIPA lysis buffer, Beyotime, Shanghai, China) into a 12% Tris-Gly SDS-PAGE gel (WSHT, Shanghai, China), with 10 μg of protein per well. The gel was run at 80 V for the stacking phase and 110 V for the separating phase. After electrophoresis, the proteins were transferred to a PVDF membrane in an ice bath at a constant current of 250 mA. Once the transfer was complete, the membrane was blocked in a solution containing 5% non-fat dry milk (Solarbio, Beijing, China) for 1 h. It was then incubated overnight at 4°C with the primary antibodies: CD9 (D8O1A) rabbit mAb (Cell Signaling Technology, Danvers, MA, United States), CD63 (E1W3T) rabbit mAb (Cell Signaling Technology, Danvers, MA, USA), recombinant anti-TSG101 antibody (Abcam, Cambridge, UK), and recombinant anti-calnexin antibody (Abcam, Cambridge, UK), all diluted 1:1000 on a shaking platform. After three washes using TBST (Servicebio, Wuhan, China), the membrane was incubated for 1 h at 4°C with the goat anti-rabbit IgG (ZSGB, Beijing, China) diluted 1:5000 on a shaking platform. Following three additional washes with TBST, the membrane was exposed and developed using a chemiluminescence imaging analysis system (Tanon 4600, Shanghai, China).

### 2.5 Transmission electron microscopy and nanoflow cytometry analysis

For transmission electron microscopy (TEM) analysis, exosome samples were initially fixed with a 2.5% glutaraldehyde solution at 4°C for at least 12 h to preserve their structural integrity. Following fixation, a 5–10 μL aliquot of the exosome suspension was applied to a copper grid and allowed to air-dry at room temperature for 5 min. Any excess liquid was then wicked away using filter paper. The grids, now containing the exosome samples, were stained with saturated uranyl acetate for 1 min to enhance contrast and visualize the exosomal membrane. After staining, the grids were air-dried to remove any residual staining solution. The prepared grids were examined using a Tecnai G2 Spirit TEM (FEI, Hillsboro, Oregon, United States) operated at an acceleration voltage of 80 kV to obtain high-resolution images of the exosomes.

We used the nanoflow device (U30, Fuliu, Xiamen, China) to detect exosomes derived from iPSCs, strictly following the instructions provided in the manual. Initially, specialized quality control standards, including particle concentration and particle size standards, were used to validate the device’s performance. During the quality control process, the device was preheated, and the standards were sequentially loaded while data were recorded to ensure that the measurement results fell within acceptable ranges. Following successful quality control, the extracted iPSC exosome samples were diluted to an appropriate concentration and loaded into the device for detection. Suitable detection parameters were set, and data were recorded in real time. Subsequently, the corresponding software was used to analyze the particle size distribution and concentration of the exosomes.

### 2.6 Detection of AmB content

Following dialysis, the samples were extracted from the dialysis bags and diluted with DMSO. AmB concentration standards were prepared using the same solvent as the samples. The standards and the samples to be analyzed were sequentially added to a microplate. The detection of AmB content was performed using a microplate reader at a wavelength of 405 nm. The amount of AmB loaded into the exosomes was calculated based on the concentration curve derived from the standards.

$\begin{array}{l} \mathrm{Drug loading efficiency}\;\left(\%\right)\\ =\left(\mathrm{mass of}\;AmB\;\mathrm{loaded into exosomes}/\mathrm{initial mass of}\;AmB\;\mathrm{added}\right)\\ \times 100 \end{array}$

Drug loadingμgAmB/10^9particles=mass ofAmB/number of exosome particles×10^9.

### 2.7 Drug-loading methods for incorporating AmB into iPSC-Exos

We compared four drug-loading methods: co-incubation, ultrasound, extrusion, and electroporation. Each method used a sample volume of 1 mL, containing iPSC-Exos at a concentration of 50 μg/mL and AmB at a concentration of 10 μg/mL. Finally, the absorbance (optical density, OD) of each well was measured at 450 nm using a microplate reader (Varioskan LUX, Thermo Fisher Scientific, Waltham, MA, United States). Detailed descriptions of each method are provided in Table 1.

**Table 1:** Drug-loading methods for incorporating AmB into iPSC-derived exosomes.

Drug-loading methods	Condition parameters
Co-incubation	Incubate at 37°C in the dark for 2 h
Electroporation	Perform electroporation using the Lonza Amaxa 4D Nucleofector with program CA137, followed by incubation at 37°C in the dark for 1 h
Extrusion	Utilize the Avanti Lipids extruder with a 100-nm pore-size PC membrane, extruding 20 times, followed by incubation at 37°C in the dark for 1 h
Ultrasound	Apply 37 kHz, 30% power, pulse sonication for 15 min, followed by incubation at 37°C in the dark for 1 h

### 2.8 Cytotoxicity evaluation of iPSC-Exo/AmB

We used the CCK-8 assay to evaluate the proliferation of HEK 293 T and RAW264.7 cells. The CCK-8 kit was purchased from Beyotime (Shanghai, China) and used according to the manufacturer’s instructions. The cells were seeded in 96-well plates during the exponential growth phase at a density of 2 × 10^4 cells per well and treated with various concentrations of amphotericin B and iPSC-Exo/AmB based on the experimental groups. The cells were cultured for 6 h in a 37°C incubator with 5% CO2. At the end of the culture period, cell morphology was observed under a microscope. Subsequently, 10 μL of 5 mg/mL CCK-8 reagent was added to each well and incubated for 1 h in the dark at 37°C with 5% CO2.

In addition, we utilized two cell viability and cytotoxicity detection kits from Beyotime (Shanghai, China) and BestBio (Shanghai, China) to assess cell survival and cytotoxicity. The cells were treated according to the experimental groups and then incubated with calcein AM (1:1000), propidium iodide (PI) (1:1000), and Hoechst 33342 (1:100) in serum-free medium at 37°C for 20–30 min. After washing three times with serum-free medium to remove unbound dye, a complete growth medium was added, and fluorescence images were captured to evaluate cell survival. For apoptosis detection, the cells were washed with PBS, briefly digested with trypsin (without EDTA), and centrifuged at 1000 rpm for 5 min. They were then resuspended in pre-cooled 1× PBS (4°C) and washed again. Following this, the cells were suspended in 300 μL of 1× binding buffer, incubated with 5 μL of Annexin V-FITC in the dark for 15 min, and stained with 10 μL of PI for an additional 10 min. Finally, the samples were analyzed using a flow cytometer.

### 2.9 Biochemical assessment of nephrotoxicity and hepatotoxicity in C57BL/6 J mice

C57BL/6 J mice were administered AmB, iPSC-Exo/AmB, or iPSC-Exo at a dose of 0.25 mg/kg. Blood samples were collected 48 h post-treatment to assess biochemical parameters. Serum samples were obtained from the mice, and biochemical markers such as blood urea nitrogen (BUN), creatinine (CRE), uric acid (UA), and urinary protein (UP) levels were measured to evaluate nephrotoxicity. Hepatotoxicity was assessed by measuring the levels of aspartate aminotransferase (AST), alanine aminotransferase (ALT), direct bilirubin (DBIL), and total bilirubin (TBIL) in the serum. All analyses were performed in triplicate using commercial assay kits purchased from Jiancheng Bioengineering Institute (Nanjing, China), following the manufacturer’s instructions.

### 2.10 Antifungal susceptibility testing of iPSC-Exo/AmB *in vitro*

The minimum inhibitory concentration (MIC) assay was conducted to evaluate the sensitivity of antifungal drugs, as described by Liu et al. (2020). The MIC values for iPSC-Exo/AmB and AmB were determined using the broth microdilution method, following the Clinical and Laboratory Standards Institute (CLSI) guideline M27-A3. A series of diluted iPSC-Exo/AmB and AmB solutions were mixed with RPMI 1640 liquid culture medium at 30°C, resulting in a final yeast suspension of 1 × 10^3 cells/mL in a 96-well microtiter plate. For Candida species, the MIC was measured after 24 h of incubation, while for other fungi, it was determined after 72 h. Each antifungal susceptibility test included the quality control strain *C. parapsilosis* ATCC 22019 to ensure assay accuracy. The MIC range of fluconazole against *C. parapsilosis* ATCC 22019 was found to be between 0.5 μg/mL and 4 μg/mL, confirming that the test results were within an acceptable range.

### 2.11 Assessing the therapeutic effects of iPSC-Exo/AmB *in vivo*

To evaluate the therapeutic effects of drugs on CM, this study utilized female C57BL/6 J mice aged 6–8 weeks. A systemic infection model was established by intravenously injecting 200 μL of a suspension containing the viable *C. neoformans* H99 strain (1 × 10^6 CFU) via the lateral tail vein. Two hours post-infection, the mice were treated with AmB, iPSC-Exo/AmB, or iPSC-Exos at a dose of 0.25 mg/kg (iPSC-Exos were used at the same particles), while untreated mice served as controls. At 48 h after infection, the mice were euthanized, and brain tissues were collected for fungal burden measurement and periodic acid–Schiff (PAS) staining. The serum was harvested, and commercial assay kits procured from Jiangsu Sumeike Biological Technology (Yancheng, China) were used to measure the concentrations of tumor necrosis factor-alpha (TNF-*α*) and interleukin-6 (IL-6) according to the manufacturer’s instructions. All experiments were performed in triplicate to ensure the accuracy and reproducibility of the results.

### 2.12 Biodistribution of iPSC-Exo/AmB and AmB *in vivo*

To investigate the biodistribution of iPSC-Exo/AmB and AmB in C57 BL/6 J mice, we administered a 1 mg/kg injection and sacrificed the mice at 1, 3, 6, 9, and 12 h post-injection (*n* = 3). The kidneys, liver, and brain tissues were harvested and processed with acetonitrile for ultrasonic homogenization at 70 Hz for 2 min. The samples were then centrifuged at 13,000 r/min for 15 min at 4°C, and the supernatant was filtered through a 0.22-μm membrane.

Sample analysis was conducted using a solid-phase chromatography column (Waters HSS T3, 2.1 × 100 mm, 1.8 μm, Waters, United States) coupled with ultra-performance liquid chromatography (UPLC) and tandem mass spectrometry (MS/MS, AB Sciex 4,000 QTRAP, United States). The chromatographic conditions included a column temperature of 30°C and a flow rate of 0.3 mL/min, using a gradient elution process. The mobile phase began with 95% ultrapure water and 5% acetonitrile, adjusted to 40% ultrapure water and 60% acetonitrile at 1.00 min, and finally transitioned to 5% ultrapure water and 95% acetonitrile at 3.00 min.

### 2.13 Statistical analysis

The data were statistically analyzed using SPSS 23.0 software. Data normality was assessed using the Kolmogorov–Smirnov test. For normally distributed data, numerical variables are presented as mean ± standard deviation (x ± s). One-way analysis of variance (ANOVA) was used for group comparisons, and pairwise comparisons between groups were conducted using the least significant difference (LSD) *t*-test. A *p*-value of less than 0.05 was considered statistically significant.

## 3 Results

### 3.1 Extraction and identification of iPSC-Exos

**Figure 1:**
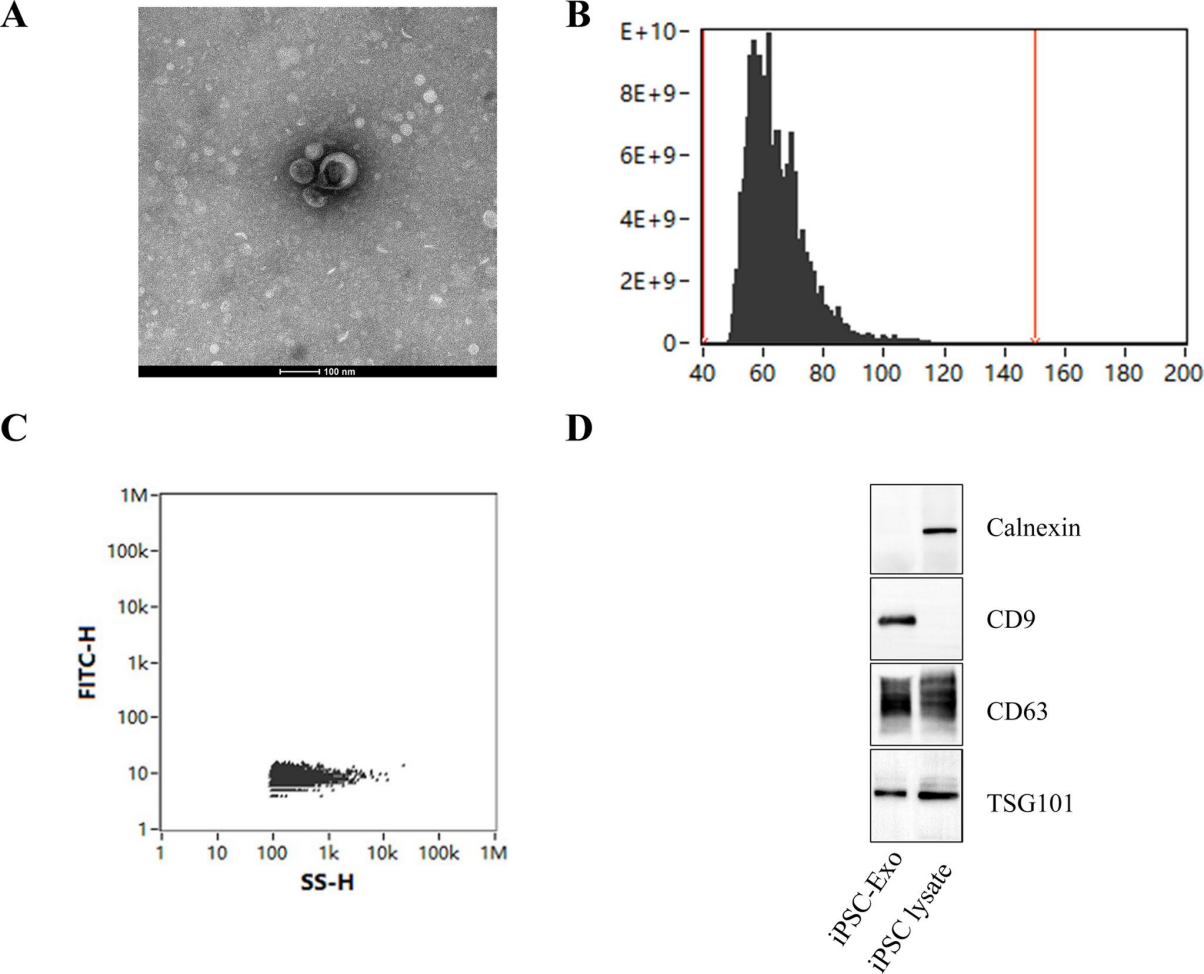
Characterization of iPSC-derived exosomes. **(A)** Transmission electron microscopy (TEM) images of iPSC-Exos, illustrating their characteristic cup-shaped morphology with well-defined edges. Scale bar = 100 nm. **(B,C)** Nanoflow cytometry (nFCM) analysis displaying the particle size distribution of exosomes, which reveals a median diameter of 62.75 nm and an average particle concentration of 3.03E+11 particles/mL. **(D)** Western blot analysis confirms the presence of exosomal markers CD9, CD63, and TSG101. The absence of calnexin further indicates the purity of the exosome preparation (see Supplementary Figure S1 for the original images of the western blot).

Exosomes were successfully extracted from iPSCs using ultrafiltration and ultracentrifugation techniques. TEM revealed that these exosomes possess a distinct membrane structure and exhibit typical disk-like morphology with well-defined edges (Figure 1A). Further analysis using nanoflow cytometry (nFCM) indicated that the extracted exosomes have a median diameter of 62.75 nm, an average diameter of 64.49 nm, and a standard deviation of 9.34 nm (Figure 1B), with an average particle concentration of 3.03E+11 particles/mL (Figure 1C). Western blotting confirmed the presence of the exosomal marker proteins CD9, CD63, and TSG101, while the endoplasmic reticulum protein calnexin was not detected (Figure 1D). These results demonstrate that the iPSC-Exos conform to the standard characteristics of exosomes, providing a solid foundation for subsequent experimental research.

### 3.2 Determination of the optimal feeding concentration of AmB

In this study, we investigated the hydrophobicity of AmB and evaluated its loading efficiency and purification process in nanodrug carriers. We referenced the preparation and purification methods of AmB described in relevant patents (CN108714151 B and CN111529506 A). A 48-h dialysis experiment was conducted at 25°C using a 14 kDa dialysis bag with three concentrations of AmB: 20, 10, and 5 μg/mL. The results showed that the residual rate of AmB at a concentration of 20 μg/mL was 19.5%, while the residual amounts at 10 μg/mL and 5 μg/mL were below the detection limit (as shown in Table 2). Based on these findings, we determined that 10 μg/mL is the optimal feeding concentration for AmB. This concentration not only enhances loading efficiency but also facilitates subsequent purification steps, thereby improving the overall efficiency and purity of the preparation process.

**Table 2:** Relationship between dialysis clearance rate and concentration of free AmB.

Feed concentration (μg/mL)	Residual after dialysis (μg/mL)	Residual ratio (%)^b^
20	3.9	19.5%
10	< LOD^a^	N/A
5	< LOD	N/A

a LOD: Limit of detection.

b Residual ratio (%) = (residual amphotericin B concentration after dialysis/amphotericin B feed concentration) * 100.

### 3.3 Co-incubation: the optimal choice for efficient AmB loading into iPSC-Exos

**Figure 2:**
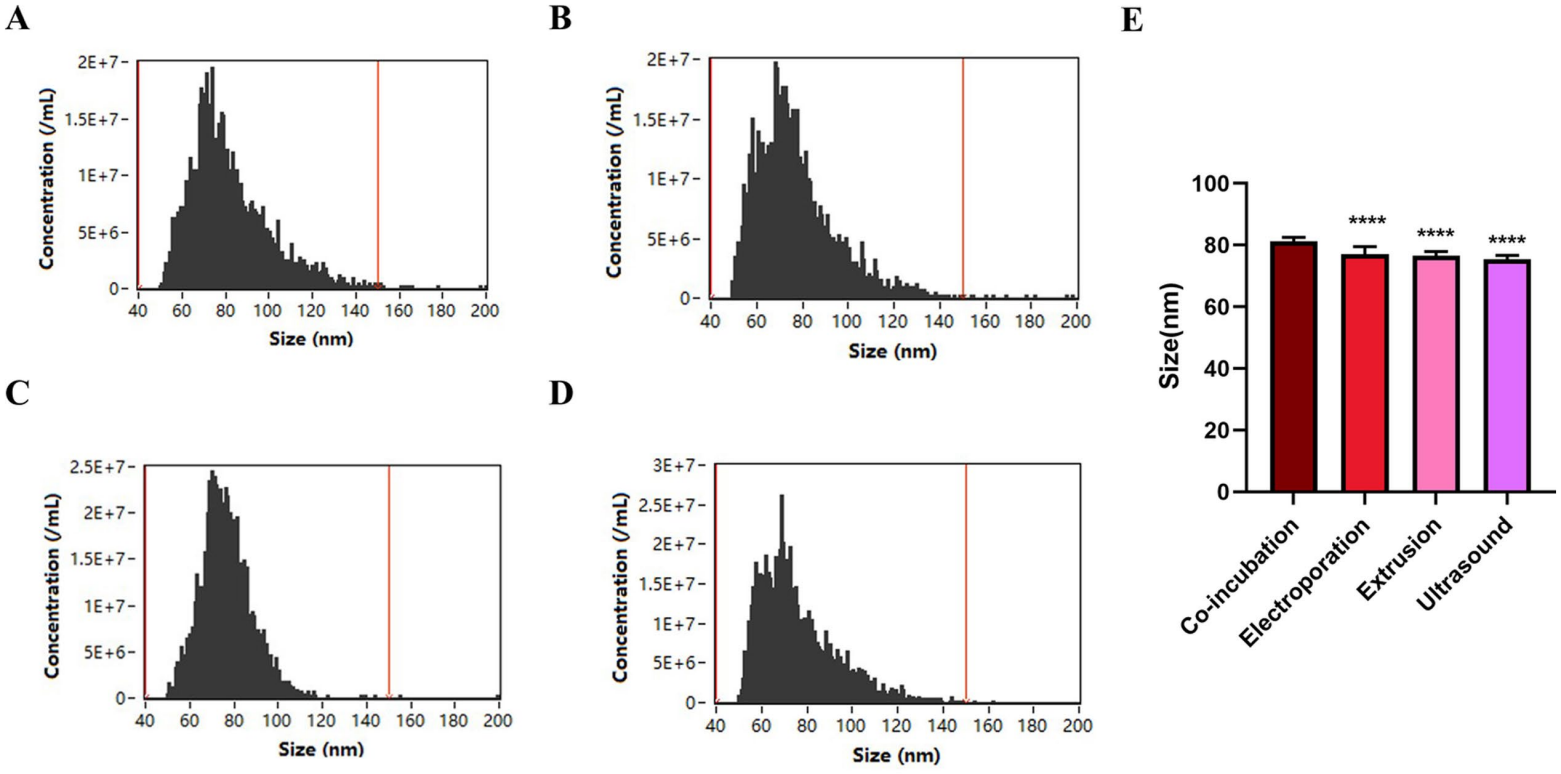
Particle size distribution of iPSC-derived exosomes loaded with amphotericin B using different methods. **(A)** Co-incubation—this method results in particles with a mean diameter of 81.31 nm and a median diameter of 77.75 nm. **(B)** Electroporation—electroporation leads to particles with a mean diameter of 77.01 nm and a median diameter of 74.25 nm. **(C)** Extrusion—using a 100-nm pore-size membrane, extrusion results in particles with a mean diameter of 76.66 nm and a median diameter of 75.75 nm. **(D)** Ultrasound—applying 37 kHz pulse sonication, this method achieves particles with a mean diameter of 75.45 nm and a median diameter of 71.75 nm. **(E)** Comparison of particle size distribution of iPSC-derived exosomes loaded with amphotericin B after different drug-loading methods. *p < 0.05, **p < 0.01, ***p < 0.001, ****p < 0.0001.

To enhance the drug loading capacity and efficiency of iPSC-Exos for AmB, we evaluated four drug-loading methods: co-incubation, ultrasound, extrusion, and electroporation. Figure 2 displays the particle size distribution post-treatment as measured by nFCM, facilitating a comparative analysis of these drug delivery strategies. Our analysis revealed that all methods increased particle size, with co-incubation showing a significant median diameter increase of 15 nm (*p* < 0.0001). Co-incubation not only elevated the drug loading efficiency to 43.6% but also achieved a substantial loading capacity of 4.6 μg AmB per 10^9 particles, as reported in Table 3. This enlargement exceeds the renal excretion threshold, suggesting that the iPSC-Exo/AmB complex formed via co-incubation could significantly reduce AmB accumulation in the kidneys, thereby potentially mitigating its nephrotoxic effects.

**Table 3:** Assessing the performance of different amphotericin B-loading methods in iPSC-derived exosomes.

Drug-loading method	Mean (nm)	Median (nm)	Std. deviation (nm)^a^	Drug concentration (μg/mL)	Loading efficiency (%)	Drug loading (μg AmB/10^9 particles)
Co-incubation	81.31	77.75	17.14	4.4	43.6	4.6
Electroporation	77.01	74.25	16.47	2.6	26.4	2.6
Extrusion	76.66	75.75	10.89	2.9	29.1	2.7
Ultrasound	75.45	71.75	16.44	3.7	37.2	3.3

a Std. deviation: standard deviation.

### 3.4 Protective effects of iPSC-Exo/AmB against AmB-induced cytotoxicity

**Figure 3:**
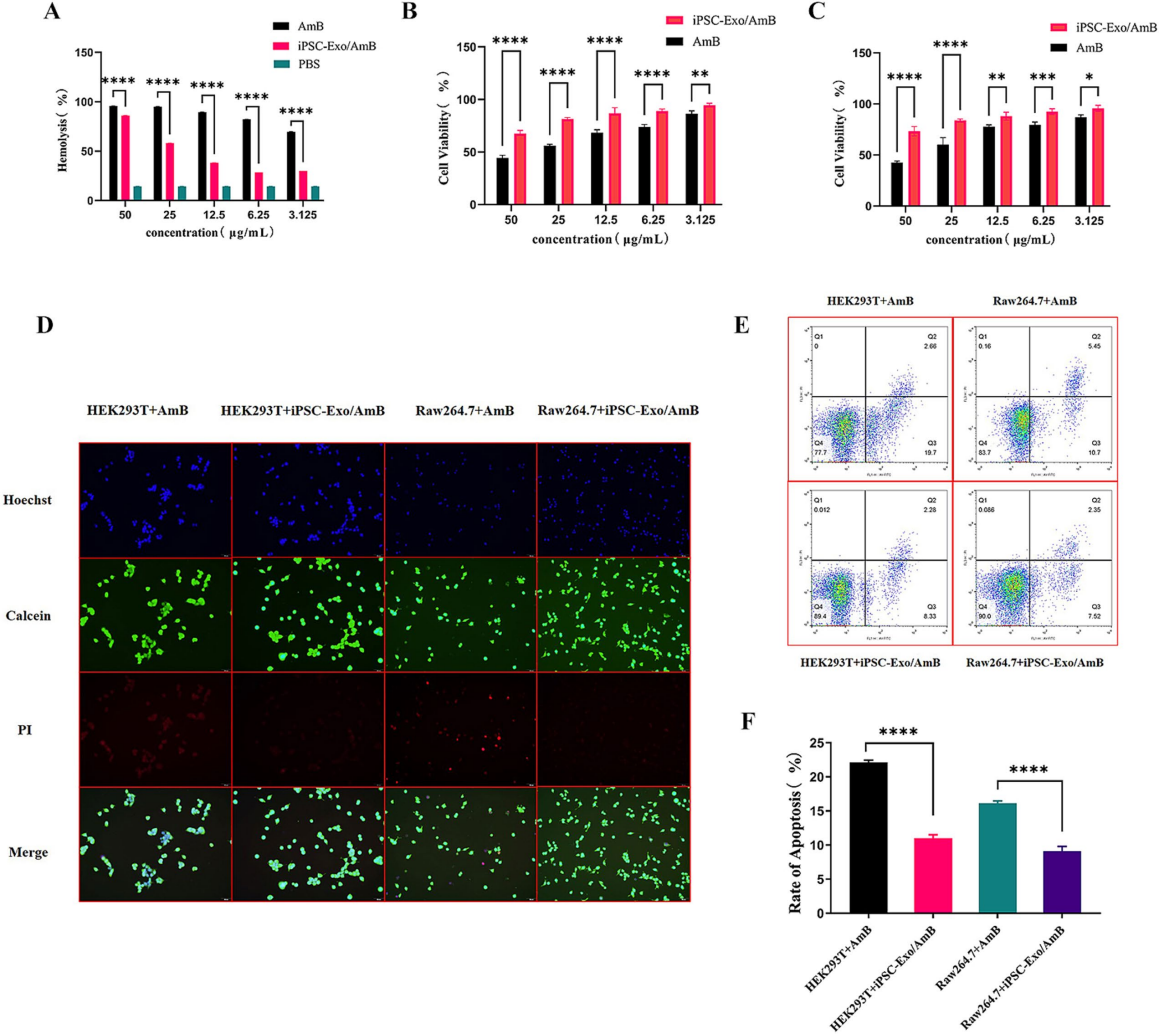
Cytotoxicity and apoptosis analysis of iPSC-Exo/AmB. **(A)** The hemolysis activity assay demonstrates that the iPSC-Exo/AmB complex significantly reduces hemolytic activity at all tested concentrations compared to AmB. This finding indicates a lower destructiveness to rabbit red blood cells. **(B,C)** The CCK-8 assay results for HEK293T and RAW264.7 cells confirm the dose-dependent toxicity of AmB. In addition, these results highlight the protective effect of iPSC-Exo/AmB against AmB-induced cytotoxicity. **(D)** Live–dead cell staining of HEK293T and RAW264.7 cells treated with AmB and iPSC-Exo/AmB at a concentration of 25 μg/mL illustrates the reduced cytotoxicity associated with iPSC-Exo/AmB. **(E,F)** Flow cytometry analysis—flow cytometry analysis of apoptosis in HEK293T and RAW264.7 cells treated with AmB and iPSC-Exo/AmB at a concentration of 25 μg/mL shows a significant decrease in the rate of cell apoptosis with iPSC-Exo/AmB treatment. *p < 0.05, **p < 0.01, ***p < 0.001, ****p < 0.0001.

Based on the preceding research, we obtained iPSC-Exos at a concentration of 3.03E+11 particles/mL. Co-incubation method revealed that each 10^9 particles were found to carry 4.6 μg of AmB. We then dissolved the iPSC-Exo/AmB in PBS and diluted it to prepare a stock solution with a concentration of 50 μg/mL of AmB. AmB is known to interact with cholesterol-containing cell membranes, leading to damage in host cells. As shown in Figure 3A, our hemolysis activity test results indicated that iPSC-Exo/AmB significantly reduced hemolytic activity compared to AmB at all tested concentrations (*p* < 0.0001), demonstrating lower destructiveness to rabbit red blood cells.

The CCK-8 assay results, as presented in Figures 3B,C, revealed the dose-dependent toxicity of AmB on HEK293T and RAW264.7 cells. At a concentration of 25 μg/mL, the viability of HEK293T cells decreased to 60.25%, while RAW264.7 cells exhibited a viability of 56.11%. Increasing the AmB concentration to 50 μg/mL further reduced viability to 42.66 and 44.29%, respectively, highlighting the significant inhibitory effect of high AmB concentrations. In contrast, cells treated with iPSC-Exo/AmB displayed significantly higher viability at the same concentrations (*p* < 0.05). At 25 μg/mL, the viability of HEK293T and RAW264.7 cells was maintained at 73.32 and 81.44%, respectively. At 50 μg/mL, these rates were 73.32% for HEK293T cells and 67.56% for RAW264.7 cells. These findings indicate that iPSC-Exo/AmB effectively mitigates the cytotoxic effects of AmB.

In addition, at the working concentration of 25 μg/mL, live–dead cell staining (Figure 3D) and flow cytometry (Figures 3E,F) results demonstrated that iPSC-Exo/AmB significantly reduced the rate of cell apoptosis. Collectively, these results suggest that iPSC-Exo/AmB offers significant advantages over traditional AmB in reducing toxicity to HEK293T and RAW264.7 cells, thereby validating its safety and potential as an antifungal agent.

### 3.5 iPSC-Exo/AmB mitigates the adverse effects of AmB on liver and kidney function

**Figure 4:**
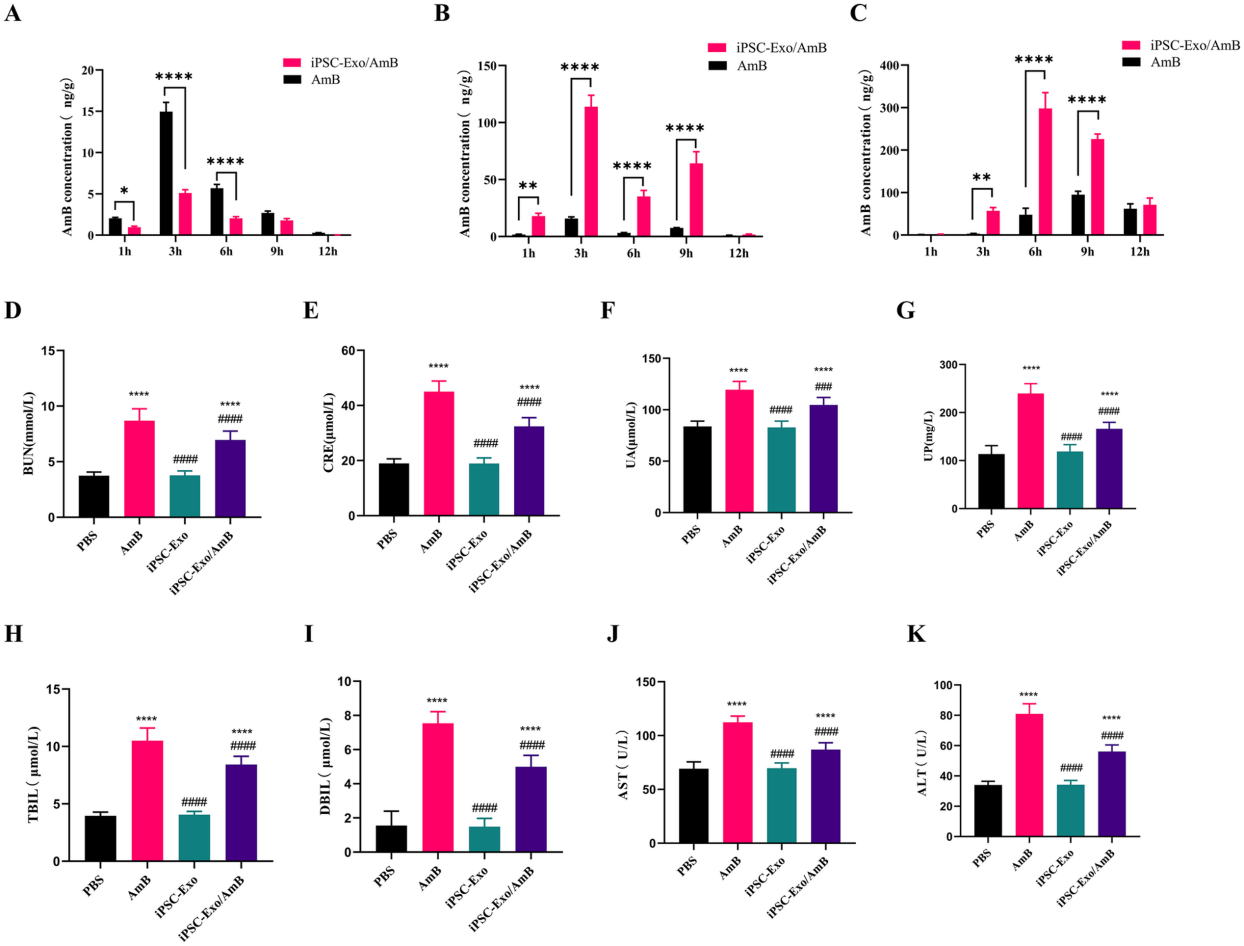
Assessment of AmB biodistribution, nephrotoxicity, and hepatotoxicity in C57BL/6 J mice treated with iPSC-Exo/AmB. **(A–C)** UPLC-MS/MS analysis of AmB concentration in the kidneys **(A)**, liver **(B)**, and brain **(C)** of C57BL/6 J mice following intravenous injection of AmB or iPSC-Exo/AmB at a dose of 1 mg/kg at specified time points. **(D–K)** Biochemical analysis of serum samples from C57BL/6 J mice treated with AmB, iPSC-Exo/AmB, or iPSC-Exo at a dose of 0.25 mg/kg. The levels of various biochemical markers are displayed, including blood urea nitrogen (BUN), creatinine (CRE), uric acid (UA), urinary protein (UP), aspartate aminotransferase (AST), alanine aminotransferase (ALT), direct bilirubin (DBIL), and total bilirubin (TBIL). Mice treated with iPSC-Exo/AmB exhibited significantly lower levels of these markers compared to those treated with AmB alone, indicating reduced nephrotoxicity and hepatotoxicity. Compared to the control group: *p < 0.05, **p < 0.01, ***p < 0.001, ****p < 0.0001, compared to the AmB treatment group: #*p* < 0.05, ##*p* < 0.01, ###*p* < 0.001, ####*p* < 0.0001.

AmB is often limited in clinical applications due to its significant nephrotoxicity. In this study, we developed iPSC-Exo/AmB, with a particle size of approximately 80 nm, which exceeds the renal excretion threshold but remains within the hepatic excretion range (Liu et al., 2020). To elucidate the *in vivo* distribution of the drug, we used the UPLC-MS/MS method to compare the concentrations of AmB in the liver and kidneys of mice after intravenous administration of AmB and iPSC-Exo/AmB (see Figures 4A,B). The results indicated that both formulations achieved their maximum concentrations at 3 h. Notably, compared to AmB alone, the administration of iPSC-Exo/AmB resulted in significantly higher drug concentrations in the liver and significantly lower concentrations in the kidneys, with a relatively slow and weak accumulation process observed in the latter.

To evaluate the safety and efficacy of iPSC-Exo/AmB, we used C57BL/6 J mice and administered AmB, iPSC-Exo/AmB, or iPSC-Exo at a dose of 0.25 mg/kg (with iPSC-Exos used at the same particle concentrations). After 48 h, we collected blood samples to measure various biochemical indicators. The results showed that (Figures 4D–K), compared to the PBS control group, mice treated with AmB had significant increases in BUN, CRE, UA, UP, AST, ALT, DBIL, and TBIL, indicating adverse effects on liver and kidney functions. In contrast, the iPSC-Exo group showed no significant changes, suggesting that iPSC-Exo does not burden these organs. Moreover, mice treated with iPSC-Exo/AmB exhibited significant reductions in BUN, CRE, UA, UP, AST, ALT, DBIL, and TBIL compared to the AmB-only group, although iPSC-Exo/AmB is more widely distributed in the liver (Figure 4B). These findings indicate that the iPSC-Exo/AmB complex effectively mitigates the adverse effects of AmB on liver and kidney functions.

### 3.6 Antimicrobial efficacy and therapeutic potential of iPSC-Exo/AmB against *Cryptococcus neoformans*

In this study, we assessed the minimum inhibitory concentrations (MICs) of traditional AmB and iPSC-Exo/AmB against *C. neoformans*, including the standard strain H99 and clinical isolate, through *in vitro* experiments. The microbroth dilution method was used, adhering strictly to the CLSI M27-A3 guidelines. Fluconazole’s MIC against *C. parapsilosis* ATCC 22019, which is 0.5 μg/mL, served as a quality control standard. The results (see Table 4) indicated that iPSC-Exo/AmB demonstrated at least eight times greater antimicrobial efficacy than traditional AmB in inhibiting *C. neoformans in vitro*, significantly enhancing its antimicrobial activity.

**Table 4:** MIC determination of AmB and iPSC-Exo/AmB complex against various *Cryptococcus neoformans* strains.

*Cryptococcus neoformans*	AmB (μg/mL)	iPSC-Exo/AmB (μg/mL)
Standard strain H99	1.0	0.125
Clinical strain *C. neoformans* (231016)	0.0625	<0.03125

**Figure 5:**
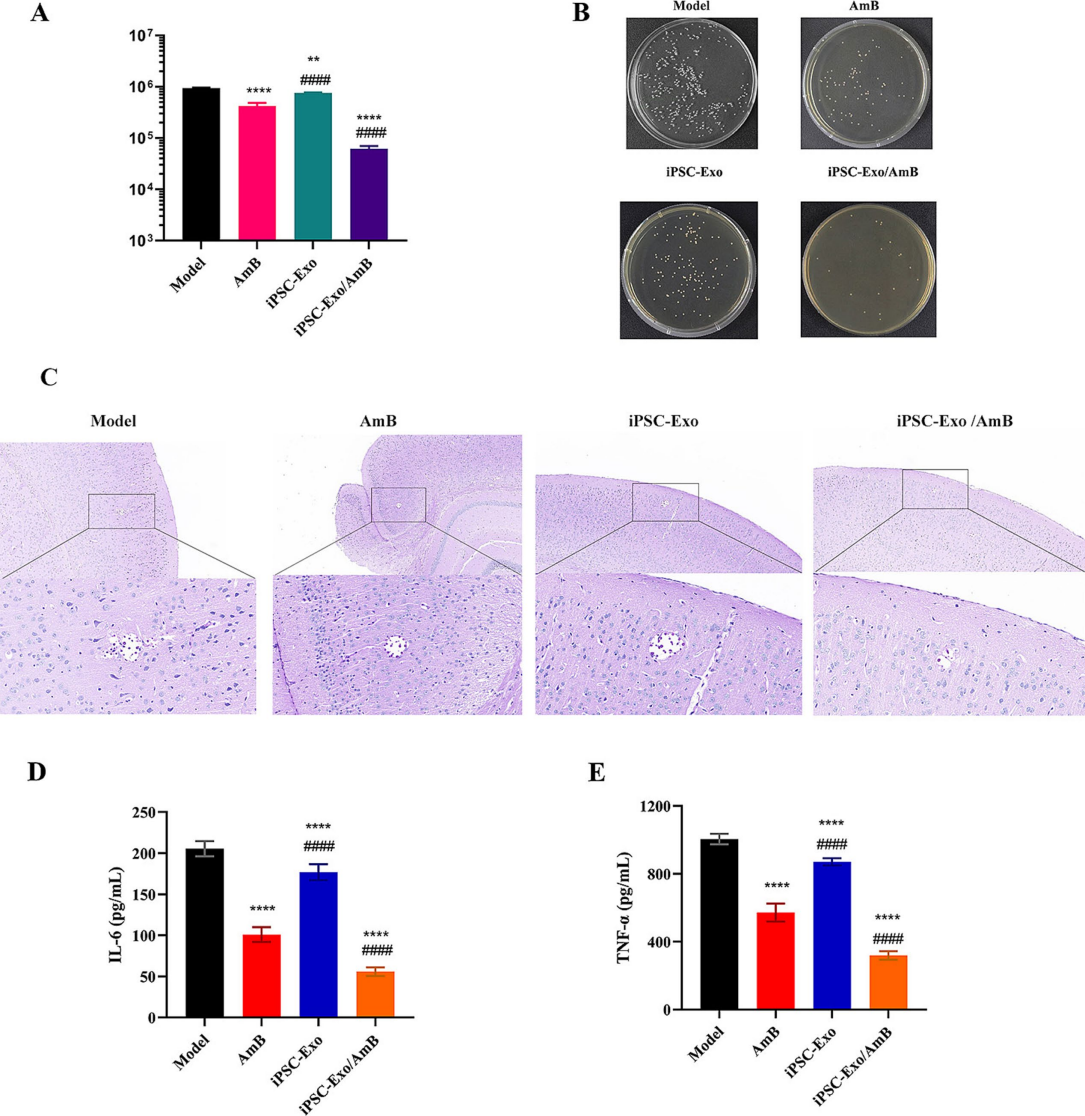
Therapeutic effects of iPSC-Exo/AmB on *Cryptococcus neoformans* infection in C57BL/6 J Mice. **(A,B)** Compared to traditional AmB treatment, iPSC-Exo/AmB treatment significantly reduced the fungal burden in the brains of mice (*p* < 0.0001), and treatment with iPSC-Exo alone also effectively reduced the fungal load (*p* < 0.01). **(C)** Periodic acid–Schiff (PAS) staining further confirmed that iPSC-Exo/AmB treatment significantly reduced the number of *Cryptococcus neoformans* in the brains of mice. **(D,E)** iPSC-Exo/AmB treatment significantly reduced the levels of tumor necrosis factor-alpha (TNF-*α*) and interleukin-6 (IL-6) in the serum of mice, indicating its significant anti-inflammatory effect. Compared to the control group: *p < 0.05, **p < 0.01, ***p < 0.001, ****p < 0.0001, compared to the AmB treatment group: #*p* < 0.05, ##*p* < 0.01, ###*p* < 0.001, ####*p* < 0.0001.

The CNS is the primary target of *C. neoformans* infection. UPLC-MS/MS analysis revealed that in C57BL/6 J mice injected intravenously with AmB and iPSC-Exo/AmB, the brain distribution of iPSC-Exo/AmB was significantly higher than that of AmB at 3 h. Moreover, at 6 h, this difference became even more pronounced, with the AmB concentration in the iPSC-Exo/AmB group increasing by approximately sixfold (Figure 4C). We established a systemic infection model in C57BL/6 J mice by intravenously administering *C. neoformans* H99 at a dose of 1 × 10^6 CFU per mouse. The mice were treated with either AmB, iPSC-Exo/AmB, or iPSC-Exo alone at a dose of 0.25 mg/kg. The mice were euthanized 48 h post-infection, and their brains were harvested for analysis. Our results indicated that the administration of iPSC-Exo/AmB significantly reduced fungal load in the brain compared to traditional AmB treatment (*p* < 0.0001). Furthermore, the administration of iPSC-Exo alone also resulted in a significant decrease in brain fungal burden compared to the model group (*p* < 0.01; see Figures 5A,B). Histological examination confirmed these findings; periodic acid–Schiff (PAS) staining of brain tissue specifically from the areas surrounding the cerebral cortex of mice treated with iPSC-Exo/AmB revealed a markedly reduced number of *C. neoformans* colonies compared to the AmB treatment group (see Figure 5C).

Although the assessment of the brain organ coefficient and changes in body weight did not reveal significant differences among the treatment groups (data not shown), the levels of inflammatory factors, such as TNF-*α* and IL-6, in the serum of mice treated with iPSC-Exo/AmB were markedly lower than those in the AmB treatment group. This suggests that iPSC-Exo/AmB may have superior anti-inflammatory effects, as illustrated in Figures 5D,E. These findings indicate that iPSC-Exo/AmB represents a novel therapeutic strategy with significant potential for treating CNS infections caused by *C. neoformans*.

## 4 Discussion

CM poses a serious medical challenge and represents a significant public health issue. According to the “2016 Global Neglected Diseases Innovation Fund (G-Finder) Report” by Policy Cures, CM is one of the most underfunded neglected diseases worldwide (Molloy et al., 2017). In October 2022, the World Health Organization (WHO) released the “Fungal Priority Pathogens List” (FPPL) to identify and highlight key fungi with unmet needs in global public health. Notably, *C. neoformans* is designated as the highest priority on this list (Casalini et al., 2024). This disease primarily affects individuals in the most economically productive age groups. Despite the availability of effective antifungal treatments, the mortality rate remains high, ranging from 30 to 100% (Zhao et al., 2023). Survivors may experience severe neurocognitive impairments, including blindness, deafness, ataxia, and epilepsy (Hamed et al., 2023; McHale et al., 2023). Currently, the selection of antifungal drugs for treating CM is extremely limited, primarily consisting of non-patented drugs that have been in use for decades and are associated with significant toxic side effects (Ngan et al., 2022).

AmB is a hydrophilic antifungal agent that remains the gold standard for treating systemic fungal infections, including CM, largely due to its low potential for developing resistance (Romani, 2012). AmB exerts its fungicidal effect by binding to ergosterol in the fungal cell membrane, which disrupts membrane stability (Campoy and Adrio, 2017). However, AmB can interact with cholesterol in human cell membranes, potentially causing damage to host cells. This interaction forms the basis for co-incubation of iPSC-Exo with amphotericin B, as the lipid bilayer of exosomes also contains cholesterol (Zhuo et al., 2024). In addition, it promotes the production of reactive oxygen species, interferes with mitochondrial function, and induces oxidative stress (Jukic et al., 2017). This action leads to the leakage of intracellular ions such as Na^+^, K^+^, H^+^, and Cl^−^, disrupting ionic homeostasis and causing damage to membranes, mitochondria, proteins, and DNA; this ultimately results in cell death (Wang et al., 2021).

Despite its efficacy, AmB has limited application due to severe toxic side effects, including infusion-related reactions (nausea, vomiting, fever, and hypoxia), kidney damage, anemia, and electrolyte imbalances, particularly hypokalemia and hypomagnesemia (Grela et al., 2018). Furthermore, AmB’s poor ability to cross the BBB poses a significant challenge in treating CM (Shao et al., 2010). Increasing the dosage to enhance drug concentration in the brain raises the risk of additional toxic effects on organ systems, particularly the kidneys, further restricting its clinical use (Wang et al., 2021).

iPSCs can be derived from various somatic cells using advanced “footprint-free” reprogramming technology. This method offers significant advantages, including diverse source materials, short induction periods, and high success rates (Huang et al., 2019; Deng et al., 2021; Park et al., 2023). Consequently, iPSCs have a wide range of applications in maintaining cellular diversity, promoting tissue repair and regeneration, and developing disease models (Paes et al., 2017; Karagiannis et al., 2019). The therapeutic effects of stem cells are primarily mediated through paracrine mechanisms in which exosomes play a critical role. Research on the mechanisms of uptake of iPSC-Exos by target cells has been extensively investigated in several studies. These studies confirm that iPSC-Exo can be effectively internalized by target cells (Li et al., 2023; Wang et al., 2023; Abbas et al., 2024). iPSC-Exos are considered promising cell-derived carriers for treating CNS diseases (Cefalo et al., 2016). This is the first study to report on the use of iPSC-Exos as carriers for treating CM. We successfully achieved efficient loading of AmB into iPSC-Exos using a co-incubation method, resulting in a drug loading efficiency of 43.6% and a loading amount of 4.6 μg AmB per 10^9 particles. Nanoflow cytometry analysis indicated that the particle size of the iPSC-Exo/AmB complex is approximately 77.75 nm, significantly below the renal excretion threshold (Liu et al., 2020). In addition, this complex demonstrated low cytotoxicity against human embryonic kidney cells, macrophages, and rabbit red blood cells. *In vivo* serum assays further revealed that iPSC-Exos significantly reduced the hepatic and renal toxicity associated with AmB. These findings strongly support the potential of iPSC-Exos as novel carriers for treating CM.

Research on mesenchymal stem cells (MSCs) and their exosomes (MSC-Exos) in the field of antifungal activity is still in its early stages. However, recent findings have revealed their significant potential in anti-inflammatory responses, immune regulation, tissue regeneration, and antimicrobial activity (Bicer, 2023). These characteristics position MSC-Exos as promising candidates for combating various microbial infections. The experimental data from this study strongly demonstrate the superiority of iPSC-Exo/AmB in antifungal efficacy. Compared to traditional AmB, the MIC of iPSC-Exo/AmB is reduced by more than eightfold, indicating enhanced efficiency in inhibiting fungal growth. Animal model experiments further support these *in vitro* findings. The study showed that the sole use of iPSC-Exos effectively reduced the fungal burden in the brains of a CM model. Furthermore, the combination treatment of iPSC-Exo/AmB significantly decreased the fungal load in the brain compared to traditional AmB therapy. These findings suggest a novel strategy for antifungal treatment.

Significant advancements have been made in elucidating the immune regulatory mechanisms associated with CM. Recent research indicates that Th1, Th2, and Th17 cytokines, along with various chemokines present in the cerebrospinal fluid of affected patients, create a complex network of immune responses (Jarvis et al., 2015; Heung and Hohl, 2019). This intricate network plays a vital role in activating macrophages, facilitating the clearance of *Cryptococcus*, and impacting the overall prognosis of the disease (Hünniger and Kurzai, 2019).

Immune reconstitution inflammatory syndrome (IRIS) has emerged as a major obstacle in the treatment of cryptococcal infections. It is closely associated with an imbalance between pro-inflammatory and anti-inflammatory responses during host immune recovery (Hünniger and Kurzai, 2019). The characteristics of IRIS include elevated levels of IFN-*γ*, the transition of macrophages to the M1 phenotype, and a shift in the immune response from anti-inflammatory to pro-inflammatory. These factors may lead to the deterioration or recurrence of symptoms in patients with CM who initially respond well to treatment (Temfack et al., 2019). Notably, IRIS is more common among HIV-infected individuals but can also affect immunocompetent hosts, and it is associated with higher mortality rates (Haddow et al., 2010).

To improve treatment outcomes for CM, the limitations of a singular antibacterial treatment strategy have become evident. Treatment approaches must consider the immune regulatory effects of antifungal medications. Our research reveals significant differences between the iPSC-Exo/AmB treatment group and the traditional AmB treatment group. Specifically, the levels of inflammatory factors, including TNF-*α* and IL-6, were significantly lower in the serum of the iPSC-Exo/AmB treatment group. This suggests a potential advantage of iPSC-Exo/AmB in anti-inflammatory activity. Although the iPSC-Exo/AmB treatment group did not show a significant advantage in improving systemic symptoms, such as weight loss and brain organ coefficients, this may be attributed to the relatively short observation period of the experiment. In the early stages of infection, the differences in these symptoms may not have reached statistical significance. As treatment continues and time progresses, we anticipate that these symptoms will gradually improve, further confirming the comprehensive benefits of iPSC-Exo/AmB in treating CM.

In high-income countries, AmBisome has emerged as the preferred treatment for CM due to its lower toxicity and improved tolerability (Chang et al., 2024). Phase III randomized controlled trials conducted in several African countries have demonstrated that a single high dose of AmBisome is non-inferior to traditional standard treatments regarding efficacy (Jarvis et al., 2022). However, AmBisome’s ability to penetrate the BBB remains limited. Its effectiveness in treating CNS fungal infections may be attributed to inflammation and local damage caused by fungal invasion, which increases BBB permeability (Takemoto et al., 2006). Furthermore, the reduced toxicity of liposomal formulations allows for potential dose escalation in treating CNS infections (Groll et al., 2000). This study investigates the use of iPSC-Exo as carriers, enhancing the ability of the iPSC-Exo/AmB to cross the BBB and significantly increasing its drug dosage within the CNS.

The anti-inflammatory and immunomodulatory effects of exosomes are significant and should not be overlooked. They effectively regulate inflammatory responses by decreasing the levels of pro-inflammatory cytokines, such as TNF-α, while releasing anti-inflammatory cytokines, such as IL-10, and promoting the release of growth factors, including BDNF, VEGF, FGF, and TGF-*β*. These findings are consistent with our research results (Zhang et al., 2020; Kim et al., 2022). In addition, iPSC-Exos facilitate the transition of macrophages from the pro-inflammatory M1 phenotype to the anti-inflammatory M2 phenotype by activating the p38 MAPK pathway and promoting mitochondrial transfer, thereby effectively controlling inflammation and enhancing tissue repair (Malekpour et al., 2023). Moreover, iPSC-Exos play a crucial role in promoting progenitor cell proliferation, angiogenesis, and extracellular matrix remodeling, which are essential for treating neurological damage caused by cryptococcal meningitis and for reconstructing the BBB (Jeske et al., 2020). They also modulate apoptosis-related factors through mitochondrial transfer, increase ATP levels, activate the PI3K/AKT signaling pathway, alleviate oxidative stress, and repair oxidative damage, all of which contribute to the improvement of cognitive function (Jeske et al., 2020; Malekpour et al., 2023).

Previous research indicates that *Cryptococcus* can internalize intact, bioactive exosomes (Rodrigues and Casadevall, 2018). We hypothesize that once *Cryptococcus* ingests the iPSC-Exo/AmB, AmB—having a tenfold greater affinity for ergosterol than for cholesterol—may be released from the exosomes, rapidly initiating its antifungal action (Walker et al., 2018). Furthermore, the uptake of exosomes may modulate *Cryptococcus’s* gene expression and signaling pathways, as well as the composition of exosomal components it releases, thereby influencing its virulence within the host (Rodrigues and Casadevall, 2018).

## 5 Conclusion

This study investigates the use of iPSC-Exos as carriers to enhance the efficacy and safety of AmB in treating CM. We successfully loaded AmB into iPSC-Exos, forming the iPSC-Exo/AmB complex, which exhibits reduced cytotoxicity and significantly mitigates AmB’s adverse effects on the liver and kidneys. The results show that, compared to traditional AmB, iPSC-Exo/AmB achieves at least an eightfold increase in antifungal efficacy *in vitro* and significantly reduces the fungal burden in the brains of mice *in vivo*. In addition, the iPSC-Exo/AmB complex demonstrates potential anti-inflammatory effects by lowering inflammatory factors in the serum. These findings suggest that iPSC-Exo/AmB, as a novel therapeutic strategy, has significant potential to effectively combat CNS infections caused by *Cryptococcus*. By reducing systemic toxicity, enhancing penetration of the BBB, and possibly exerting anti-inflammatory effects, iPSC-Exo/AmB provides new hope for treating CM and addressing refractory fungal infections in the CNS.

## 6 Limitations

Although this study demonstrates positive results in treating CM with iPSC-Exo/AmB, it has several limitations. These include the exclusive use of the C57BL/6 J mouse model, which may not fully represent human responses. In addition, the study only observed short-term effects within 48 h, without evaluating long-term treatment efficacy and safety, including survival rates and disease recurrence. The therapeutic index of the iPSC-Exo/AmB complex has not been thoroughly evaluated, particularly regarding its pharmacodynamic and pharmacokinetic properties. Furthermore, insufficient testing of various dosages restricts our understanding of the drug’s safety profile and optimal dosing regimens. There is also a lack of research on biodistribution and a limited understanding of the long-term effects on the immune system. Moreover, stricter quality control measures are needed during the preparation process, along with greater consideration of individual differences, such as genetic, environmental, and lifestyle factors. Future research should address these limitations to improve the clinical applicability of iPSC-Exo/AmB.

## Data Availability

The original contributions presented in the study are included in the article/Supplementary material, further inquiries can be directed to the corresponding authors.
