# Supplementary figures and images for: iPSC-derived exosomes as amphotericin B carriers: a promising approach to combat cryptococcal meningitis
Source: Front Microbiol. 2025 Feb 10;16:1531425. doi: 10.3389/fmicb.2025.1531425 (PMC11847882; doi:10.3389/fmicb.2025.1531425)

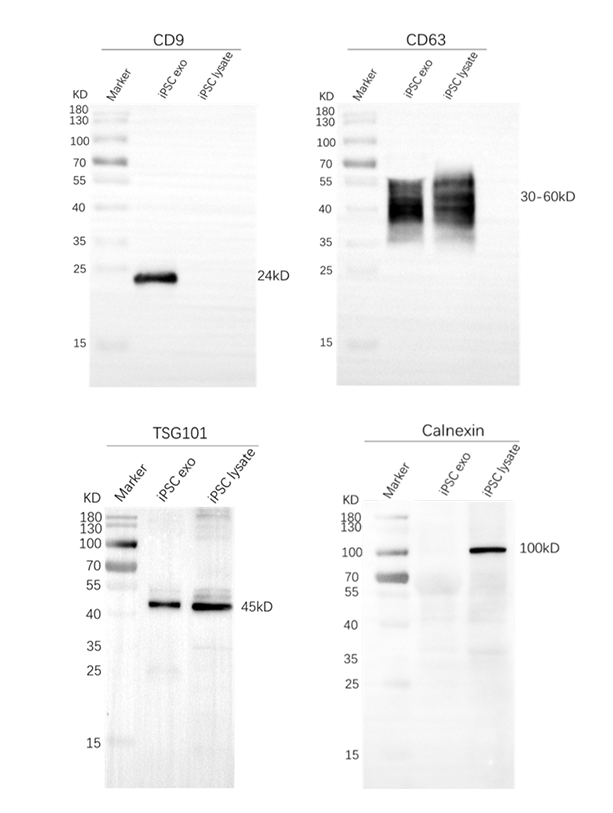

Supplement: Supplementary file 1 [file Image_1.TIF]
